# *In Silico* Discovery of Aminoacyl-tRNA Synthetase Inhibitors

**DOI:** 10.3390/ijms15011358

**Published:** 2014-01-20

**Authors:** Yaxue Zhao, Qingqing Meng, Linquan Bai, Huchen Zhou

**Affiliations:** 1 School of Pharmacy, Shanghai Jiao Tong University, 800 Dongchuan Road, Shanghai 200240, China; E-Mails: yaxuezhao@sjtu.edu.cn (Y.Z.); qqmeng@sjtu.edu.cn (Q.M.); 2 State Key Laboratory of Microbial Metabolism, Shanghai Jiao Tong University, 800 Dongchuan Road, Shanghai 200240, China; E-Mail: bailq@sjtu.edu.cn

**Keywords:** aminoacyl-tRNA synthetase, inhibitor, antibiotics, virtual screening, structure-based drug design, docking

## Abstract

Aminoacyl-tRNA synthetases (aaRSs) are enzymes that catalyze the transfer of amino acids to their cognate tRNA. They play a pivotal role in protein synthesis and are essential for cell growth and survival. The aaRSs are one of the leading targets for development of antibiotic agents. In this review, we mainly focused on aaRS inhibitor discovery and development using *in silico* methods including virtual screening and structure-based drug design. These computational methods are relatively fast and cheap, and are proving to be of great benefit for the rational development of more potent aaRS inhibitors and other pharmaceutical agents that may usher in a much needed generation of new antibiotics.

## 1. Introduction

Aminoacyl-tRNA synthetases (aaRSs) play a central role in the process of protein synthesis. They are responsible for catalyzing the attachment of the correct amino acid to its cognate tRNA through an esterification reaction at the 3′ end of tRNA. This highly specific aminoacylation reaction involves two steps:

(1) aa+ATP+aaRS⇌aaRS·aa-AMP+PPi

(2) aaRS·aa-AMP+tRNA⇌aaRS+aa-tRNA+AMP

where aa is an amino acid. The first step is formation of an aminoacyl-adenylate (aa-AMP) activated intermediate from an amino acid and ATP. During this step, ATP and the amino acid first bind the aaRS active site, and are positioned appropriately to facilitate the α-carboxylate of the amino acid to attack the α-phosphate of ATP via in-line nucleophilic displacement. In the second step, the activated amino acid is transferred from aa-AMP to the tRNA to form the aminoacyl-tRNA (aa-tRNA) via nucleophilic attack by the 2′- or 3′-hydroxyl of the 3′-terminal adenosine of the tRNA on the α-carbonyl of the aa-AMP [1].

The aaRSs are divided into two unrelated classes (class I and class II, as shown in Table 1) based on mutually exclusive sets of sequence motifs that reflect distinct active site topologies [2]. The class I synthetase active site adopts a Rossmann-fold domain and binds ATP in an extended conformation. In contrast, class II synthetase active sites are housed on an antiparallel β-fold domain, and bind ATP in a bent conformation [3]. Synthetase enzymes can be arranged into three subclasses within each class, and subclasses group enzymes that are more closely related to each other than to other enzymes in the same class [4]. Subclasses Ic and IIc contain the synthetases for aromatic amino acids, subclasses Ib and IIb comprise the synthetases for amino acids with carboxylate side chains and their amidated derivatives, and subclasses Ia and IIa include synthetases for hydrophobic amino acids.

Accurate protein synthesis and hence, cell survival, requires aaRSs to discriminate between chemically similar, non-cognate amino acids by a factor of at least 10^4^. This is difficult to achieve in one step, especially for aliphatic and hydrophobic amino acids that lack distinguishable molecular features. For example, the weakness of the additional van der Waals interactions of isoleucine compared with valine in the active site of IleRS was predicted to yield an error rate of up to one in five [5]. To overcome this problem, some aaRSs have a specific editing activity that hydrolyzes misactivated aa-AMPs (pre-transfer editing) and mischarged aa-tRNAs (post-transfer editing). This is known as the double sieve mechanism. The first sieve occurs during classical aminoacylation at the aaRS synthetic active site which binds cognate amino acids but cannot adequately distinguish between amino acids with highly similar (isosteric) or slightly smaller structures. The second sieve occurs at an editing active site which hydrolyzes non-cognate amino acids that are misactivated or mischarged. Synthetases with this additional editing site include IleRS, LeuRS, and ValRS from class I, and ThrRS, AlaRS, PheRS and ProRS from class II enzymes [6–8].

**Figure 1.**
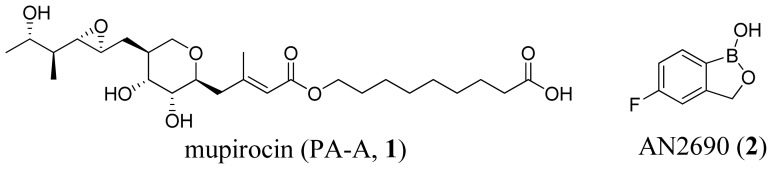
Structures of mupirocin and AN2690.

The aaRSs have become key targets for antibiotics. Inhibition of aaRSs depletes charged tRNAs, inhibits protein synthesis and leads to arrest of cell growth and ultimately cell death [9]. Inhibitors of aaRSs are being developed as antibacterials, antifungals and anti-parasitic drugs [10–13], and they also possess potent immunosuppressive activity [14]. Both synthetic and editing active sites are targets for inhibition. Mupirocin and AN2690 (Figure 1) are excellent examples of inhibitors that bind to the synthetic and editing active sites, respectively.

Mupirocin (Bactroban, GSK, London, England), a natural product of *Pseudomonas fluorescens*, is the only aaRS inhibitor approved by the US Food and Drug Administration to this date [15]. It is a mixture of several pseudomonic acids, with pseudomonic acid A (PA-A) constituting greater than 90%. Mupirocin is primarily active against gram-positive pathogens, such as *Staphylococcus aureus* and *Streptococcus pyogenes*, and is used as a topical treatment for bacterial skin infections [16]. Mupirocin is targeted against IleRS. Crystal structures of IleRS bound with mupirocin and Ile-AMP show that mupirocin binding in the IleRS synthetic site is highly similar to Ile-AMP binding [16–18]. Therefore, mupirocin is a competitive inhibitor functioning by displacing endogenous Ile and ATP.

AN2690 (Tavaborole, Anacor, Palo Alto, CA, USA) is currently in Phase 3 clinical trials for treating onychomycosis. It is a fluorinated benzoxaborole that targets LeuRS [19]. The boron atom in the oxaborole ring of AN2690 binds to both the 2′- and the 3′-hydroxyl groups on the 3′-terminal adenosine. AN2690 occupies the non-cognate amino acid binding pocket in the editing domain of LeuRS. Therefore, by trapping tRNA^Leu^ in the editing active site, such inhibitors prevent LeuRS catalytic turnover, inhibiting synthesis of leucyl-tRNA^Leu^ and consequently blocking protein synthesis.

Traditional approaches for inhibitor discovery that have proved successful include serendipity, screening natural products and known active substances to identify the active components, drug metabolites, and observing side effects of existing medicines to identify potential involvement in other pathways. In the early 1990s, combinatorial chemistry was used to synthesize huge libraries of compounds and high-throughput screening of these libraries proved particularly successful [20]. However since 2000, computational methods such as virtual screening and structure-based drug design have become more popular in pharmaceutical research. *In silico* methods save time and money in the drug discovery process [20]. Virtual screening has been widely applied in the discovery of lead compounds [21–23]. It can be divided into docking-based and pharmacophore-based procedures. A classical docking-based virtual screening approach begins with the three dimensional (3D) structure of the target protein from the Protein Data Bank (PDB) [24] or from homology modeling. Small molecule structures from commercial databases are then docked into the binding pocket of the target protein. Scoring functions are then used to evaluate and rank the binding mode of each small molecule in the target protein binding site. Finally, high scoring molecules are tested for activity in inhibition or binding assays. Currently available docking software packages for virtual screening studies are represented by Glide [25,26], Gold [27], Dock [28], and AutoDock Vina [29].

Pharmacophore features are generally represented by points in 3D space. A pharmacophore feature could be comprised of functional groups such as hydrogen bond donors, hydrogen bond acceptors, cations, anions, aromatics and hydrophobic sites [30]. Pharmacophore features can be generated by identifying common chemical features from a set of bioactive compounds, or by observing important shared interactions in protein-ligand complex structures. There are several available programs for automatic generation of pharmacophore models including Catalyst [31], Phase [32] and LigandScout [33]. The generated pharmacophore can be used to screen small molecule databases to identify appropriate compounds.

Structure-based or rational drug design is now widely applied in most stages of the drug development process, from initial hit identification to lead optimization [34,35]. Several important drugs have been developed using this method, including human immunodeficiency virus-1 protease [36] and neuraminidase [37–39] inhibitors. Central to all structure-based discovery approaches is experimental determination of the 3D structure of the target protein or protein-ligand complex, or construction of a suitably accurate homology model.

## 2. Inhibitor Identification Using Virtual Screening

### 2.1. Leucyl-tRNA Synthetase Inhibitors

**Figure 2.**
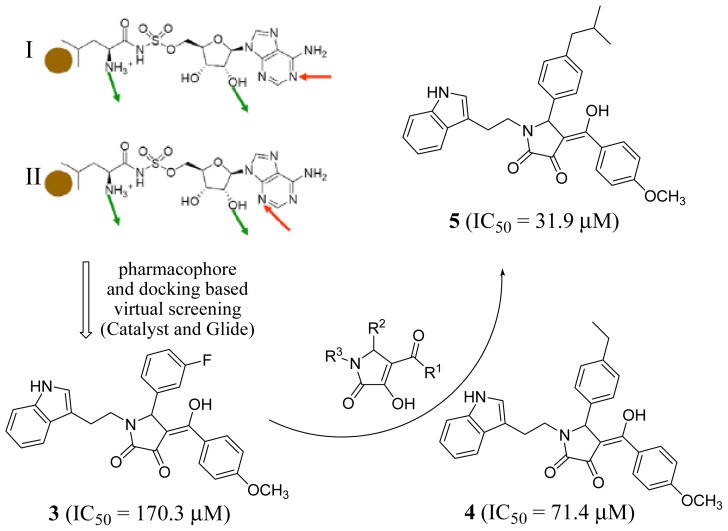
Scheme used in the identification of LeuRS inhibitors. Pharmacophores I and II: the hydrophobic site is colored as orange sphere, hydrogen bond donor is colored as green arrow, hydrogen bond acceptor is colored as red arrow.

To discover inhibitors of *Trypanosoma brucei* LeuRS in order to develop drugs against human African trypanosomiasis, Zhao *et al.* (2012) constructed a homology model of the synthetic active site based on the crystal structure of *Pyrococcus horikoshii* LeuRS (1WKB [40]) using the *in silico* mutation method [41]. By analyzing the interactions of the substrate analog Leu-AMS and *T. brucei* LeuRS, pharmacophores I and II were generated and used to screen the SPECS database [42] using Catalyst (Figure 2). Hits that matched the pharmacophores well were docked using Glide, and the 2-pyrrolinone compound **3** was identified, and found to be active *in vitro*, with an *IC*_50_ of 170.3 μM (Figure 2). Guided by the docking of compound **4** and the *T. brucei* LeuRS structure, various substituents at R^1^, R^2^, and R^3^ were designed and synthesized. Structure-activity relationship studies generally corroborated the docking model, which showed that the R^2^ phenyl group explored a new hydrophobic pocket, and the R^3^ indolyl group was essential for the favorable interaction with the leucine recognition pocket. Finally, compound **5** was identified as the most potent inhibitor (*IC*_50_ = 31.9 μM).

### 2.2. Tryptophanyl-tRNA Synthetase Inhibitors

**Figure 3.**
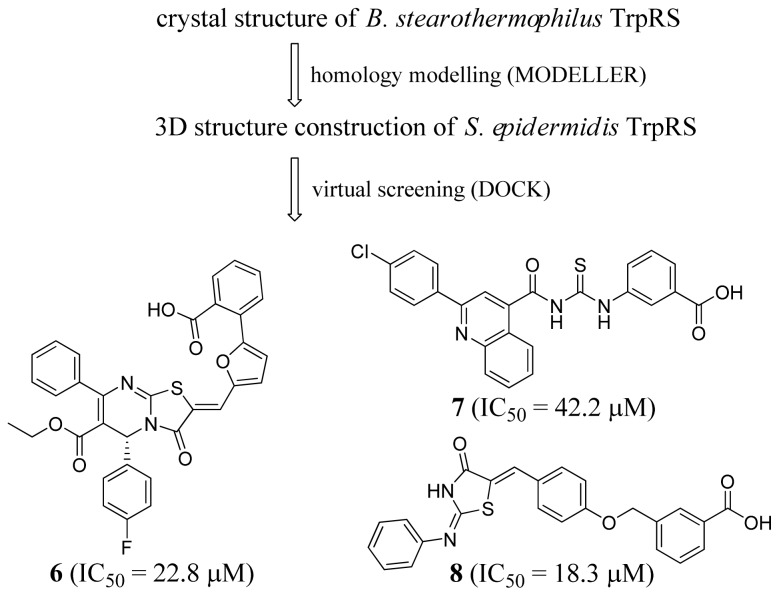
Inhibitor identification scheme of Wu *et al.* (2007) for TrpRS [43].

Wu *et al*. (2007) applied a virtual screening approach to find new lead compounds to target *Staphylococcus epidermidis* TrpRS [43]. Figure 3 shows their inhibitor identification scheme. They first constructed a homology model of *S. epidermidis* TrpRS based on the crystal structures of *Bacillus stearothermophilus* TrpRS (1MAW, 1M83, 1MAU, 1MB2 [44]) using MODELLER (Accelrys, Inc. San Diego, CA, USA). Three compounds were identified as TrpRS inhibitors that arrested *S. epidermidis* growth from the SPECS database combining virtual screening, *in vitro* and *in vivo* experiments. The *IC*_50_ values of these compounds were 22.8, 42.2 and 18.3 μM as shown using the Kinase-Glo Luminescent Kinase assay, and these results were consistent with the results of the Pyrophosphate Reagent assay. All three compounds inhibited the growth of both *S. epidermidis* ATCC 12228 and ATCC 35984 strains with micromolar minimal inhibitory concentrations (MICs) (Table 2), and also exhibited low cytotoxicity with CC_50_ > 200 μM.

### 2.3. Asparaginyl-tRNA Synthetase Inhibitors

**Figure 4.**
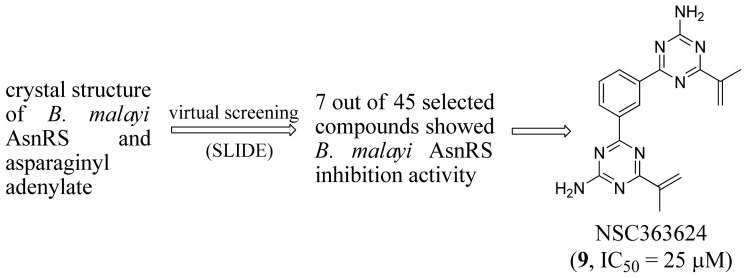
Inhibitor identification scheme of Sukuru *et al.* (2006) for AsnRS [45].

Sukuru and co-workers (2006) successfully identified seven diverse compounds that can inhibit the activity of AsnRS with micromolar affinity [45]. A template was generated using SLIDE to represent the active site of *B. malayi* AsnRS and its interactions with Asn-AMS based on X-ray crystal structure (2XGT [46]) (Figure 4). After screening the Cambridge Structural Database [47] and National Cancer Institute Plated Compounds Database [48] using SLIDE, they selected forty-five compounds for activity assays. NSC363624 is the most potent inhibitor (*IC*_50_ = 25 μM, Figure 4) which has a symmetric structure with two substituted triazine rings connected by a phenyl group.

### 2.4. Methionyl-tRNA Synthetase Inhibitors

**Figure 5.**
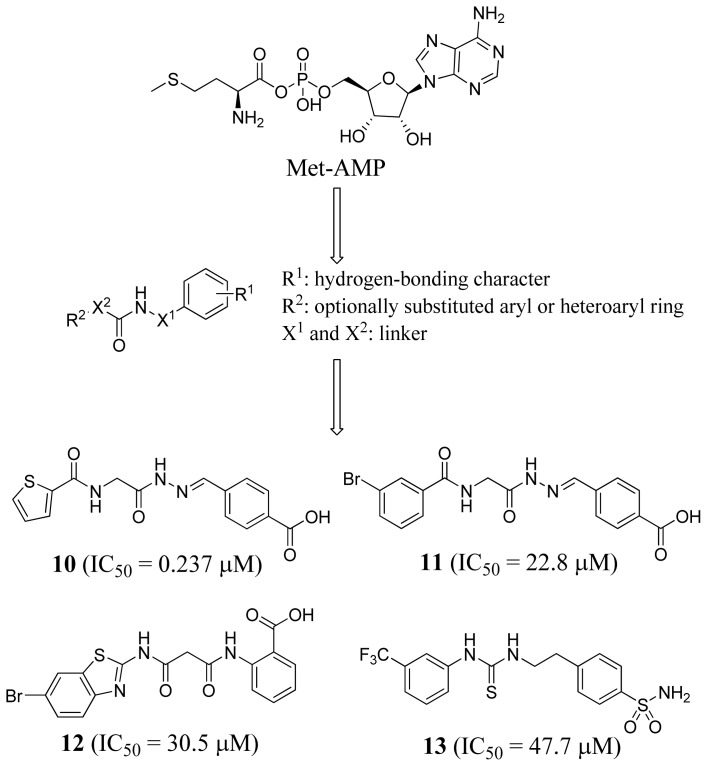
Inhibitor identification scheme of Kim *et al.* (2006) for MetRS [49].

Kim *et al*. (2006) performed a 2D-database search to discover new MetRS inhibitors [49]. Initially, they constructed a pharmacophore query based on methionyl adenylate (Figure 5) in which R^1^ has hydrogen bonding characteristics similar to those of a carboxylic acid, amide, and sulfonamide, that may act as the amine or ring nitrogen of the adenine group. R^2^ is an optionally substituted aryl or heteroaryl ring designed to mimic the lipophilic methionine side chain, and X^1^ and X^2^ are linkers. When the query structure was used to search a chemical database consisting of 508,143 commercially available compounds, four novel micromolar inhibitors of *Escherichia coli* MetRS were identified (Figure 5).

**Figure 6.**
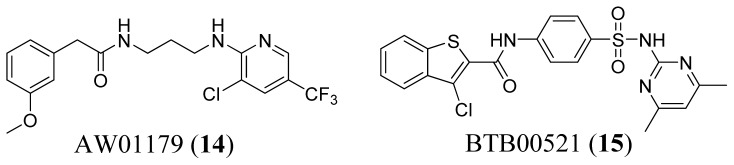
Structures of AW01179 and BTB00521.

Bharatham *et al.* (2007) identified 246 potential MetRS inhibitors, and selected 29 based on structural diversity and wide coverage of the activity range to generate pharmacophore models using CATALYST [50]. The best ranking pharmacophore model contained four chemical features including a hydrogen bond donor, a hydrophobic aliphatic substituent, and two aromatic rings. When used to search the Maybridge database [51], two inhibitors (AW01179 and BTB00521, Figure 6) were identified as the top hits, although inhibition activity was estimated using HypoGen and not tested *in vitro* or *in vivo*.

**Figure 7.**
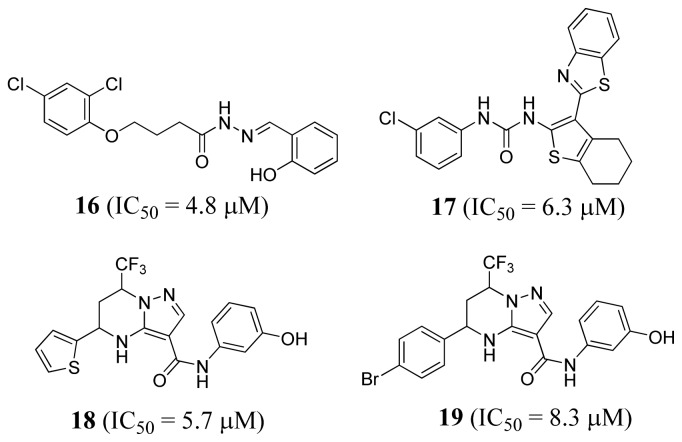
Structures of MetRS inhibitors with *IC*_50_ < 10 μM.

Finn *et al.* (2008) also attempted to identify novel MetRS inhibitors using pharmacophore-based virtual screening [52]. By analyzing crystal structures of *S. aureus* MetRS in complex with known inhibitors [52], a four point pharmacophore was generated using Catalyst which contained two hydrophobic regions, two hydrogen bond donors directed towards the carboxylate oxygens of Asp51, and an excluded volume. When this pharmacophore was used to search the ChemDiv diverse collection database [53], thirty-one compounds were identified. Enzyme assays established that twenty-two out of the thirty-one compounds demonstrated 50% or greater inhibition of *S. aureus* MetRS at 100 μM. The structures of the four most potent compounds and their *IC*_50_ values are shown in Figure 7.

## 3. Inhibitor Identification Using Structure-Based Drug Design

### 3.1. Leucyl-tRNA Synthetase Inhibitors

Ding *et al*. (2011) and Zhang *et al.* (2013) investigated *T. brucei* LeuRS using a structure-based drug design approach [12,54].

**Figure 8.**
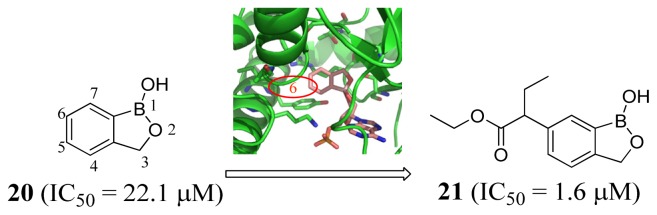
Inhibitor design scheme of Ding *et al.* (2011) for LeuRS. LeuRS is in cartoon representation and colored green, while compound **20** is in stick conformation and is colored salmon [12].

A 3D structure of the *T. brucei* LeuRS editing active site was constructed using homology modeling based on the crystal structure of *Candida albicans* LeuRS (2WFG [10]). The editing domain active site of the model was rather small and hydrophobic, and lined by nonpolar amino acid residues including Pro398, Ala443, Ile468, and Ala464. Compounds with smaller hydrophobic groups at the 6-position (compound **20**, *IC*_50_ = 22.1 μM; Figure 8) were more potent than those with hydrophilic and larger hydrophobic groups. The highest potency was shown by a compound with an ester at the 6-position (compound **21**, *IC*_50_ of 1.6 μM; Figure 8). 6-amide and 6-ketone derivatives were also developed to improve stability *in vivo*, and the 6-ketone analogs were comparable in potency to the 6-ester compounds.

**Figure 9.**
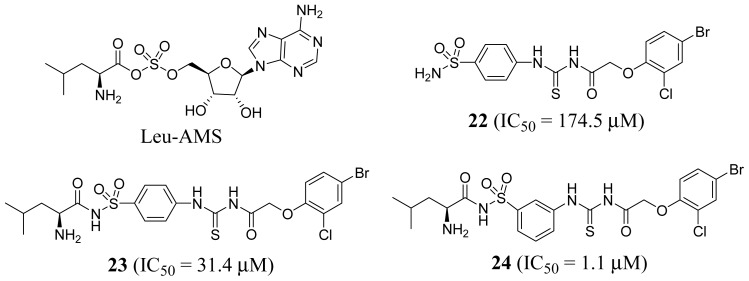
Structures of LeuRS inhibitors.

By screening an in-house database of 500 compounds, Zhang *et al.* [54] identified a *T. brucei* LeuRS inhibitor with an *N*-(4-sulfamoylphenyl)thiourea core structure (compound **22**, *IC*_50_ = 174.5 μM; Figure 9). Computational studies suggested thiourea compounds bind to the synthetic active site rather than the editing active site. In order to capitalize on the intrinsic binding affinity for the leucyl-anchoring pocket, the leucyl group was introduced (compound **23**, *IC*_50_ = 31.4 μM; Figure 9). Superimposition of the docked pose of compound **23** and Leu-AMS showed a 1,4-substitution geometry at the central phenyl ring that could lead to significant deviation. Accordingly, a 1,3-substituted analog of **23** was designed and tested, and shown to result in a more potent *T. brucei* LeuRS inhibitor (compound **24**, *IC*_50_ = 1.1 μM; in Figure 9).

### 3.2. Isoleucyl-tRNA Synthetase Inhibitors

**Figure 10.**
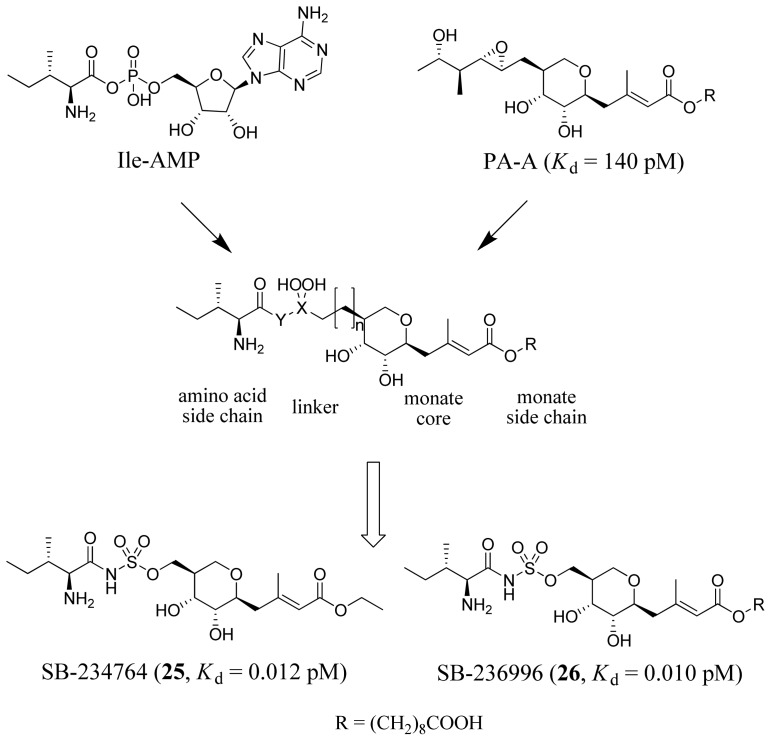
Inhibitor design scheme of Brown *et al.* (2000) for IleRS [55].

As mentioned above, mupirocin is a drug that targets IleRS. Based on the IleRS-PA-A complex crystal structure, together with a detailed understanding of the reaction cycle of IleRS and characterization of the binding mode of the Ile-AMP reaction intermediate, Brown *et al.* (2000) designed and synthesized a series of novel IleRS inhibitors (Figure 10) [55]. They noticed that the binding site of PA-A overlaps with that of Ile-AMP, such that the dihydroxytetrahydropyran and ribose rings overlap but the epoxide containing group does not occupy the Ile-binding pocket which is lined with two tryptophans. They hypothesized that extra binding energy could be achieved by appropriate introduction of an Ile moiety to improve the potency of inhibitors. Based on this, four pharmacophores (amino acid side chain, linker, monate core and monate side chain) were generated (Figure 10). Through systematic optimization of these pharmacophores, compounds SB-234764 (*K*_d_ = 0.012 pM) and SB-236996 (*K*_d_ = 0.010 pM) were developed, which were much more potent than the starting compound (*K*_d_ = 140 pM).

### 3.3. Threonyl-tRNA Synthetase Inhibitors

Teng *et al*. (2013) developed a series of potent and bacterial-selective ThrRS inhibitors through using crystal structures and structure-based drug design [56]. The crystal structure of the complex containing Thr-AMS (compound **27** in Table 2) bound to *E. coli* ThrRS was the starting point (1EVL [57]). Computational modeling using Discovery Studio suggested that compound **28** (Table 3) which has an aminoquinazoline fragment would bind to the ThrRS synthetic active site in a similar manner to Thr-AMS. Compound **28** was itself a potent inhibitor of *E. coli* ThrRS with a *K*_i_ of 2.9 nM, and the binding mode was confirmed by crystal structure. However, compound **28** also displayed potent human ThrRS inhibition activity. To explore the opportunity for selective bacterial ThrRS inhibition, compound **29** which lacks the H-bond critical for binding to Ser517 was expected to have weaker potency against human ThrRS. Indeed, **29** turned out to have a selectivity ratio of 270. Crystal structures of **29** in complex with both *E. coli* and human ThrRS demonstrated that the ATP sites of the two enzymes did bind **29** in two distinct modes.

### 3.4. Methionyl-tRNA Synthetase Inhibitors

**Figure 11.**
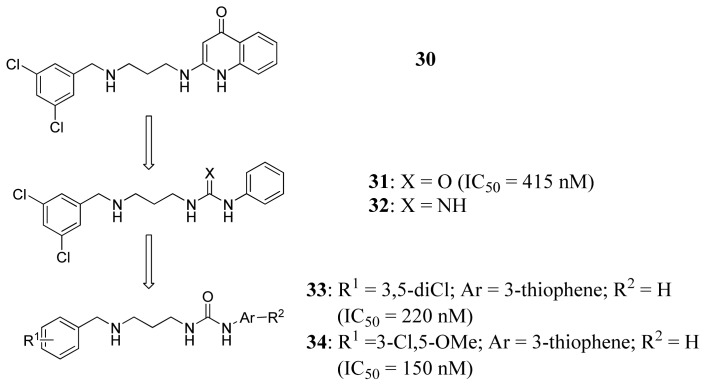
Inhibitor design scheme of Shibata *et al.* (2012) for IleRS [59].

The Buckner group have carried out extensive work on the discovery of *T. brucei* MetRS inhibitors [58–61]. Based on the crystal structure of *Clostridium difficile* MetRS [62], they built a *T. bruce*i MetRS homology model, and docking of compound **30** (Figure 11) was the starting point for inhibitor design [59]. The binding mode of **30** showed that its aminoquinolinone ring forms hydrogen bonds through its NH groups with the carboxylate of Asp287. To capitalize on this interaction, urea- and guanidine-containing analogs (compounds **31** and **32** respectively; Figure 11) were investigated. Compound **31** was chosen as a template for further exploration to develop inhibitors with better permeability properties, due to its tighter binding. By modifying R^1^, Ar and R^2^, compound **34** was developed which was significantly more potent (*IC*_50_ = 220 nM) and compound **33** had higher cell permeability (Figure 11). Additionally, compound **31** was also found to have high cell permeability and was capable of penetrating the mouse blood-brain barrier.

## 4. Conclusions

Aminoacyl-tRNA synthetases play a central role in protein synthesis by catalyzing the transfer of amino acids to their cognate tRNAs. These enzymes are clinically validated drug targets and they have been successfully targeted by anti-bacterial, anti-fungal and anti-parasitic agents. However, only one approved aaRS inhibitor is used clinically to date, illustrating the need for further aaRS inhibitor discovery and development.

All aaRSs from different species use the same reaction intermediates in the aminoacylation reaction, representing their conservatism in structures. However, some divergence has occurred throughout their evolution. For example, LeuRSs are evolved into bacterial and eukaryal/archaeal subtypes that possess a number of different residues in the active sites. Consequently, it is possible to take advantage of these active site variations to develop species-selective aaRS inhibitors, although it presents a challenge as in the development of any other selective inhibitors. The drug mupirocin which shows an 8000-fold selectivity for pathogenic IleRS over human IleRS is an excellent example of success.

Computational methods are commonly used in all areas of health science research. Among them, virtual screening and structure-based drug design have become established as powerful methods for identification and optimization of potential small molecule drugs. In this article, we reviewed the application of these approaches in the discovery and development of LeuRS, TrpRS, AsnRS, MetRS, IleRS and ThrRS inhibitors. This review demonstrates the wide use of computational methods for aaRS inhibitor discovery and development, which will surely assist the production of much needed novel antibiotics and other pharmaceutical agents.

## Figures and Tables

**Table 1.** Classes of aminoacyl-tRNA synthetases.

Class	Subclass	aaRS
I	Ia	MetRS
		ValRS
		LeuRS
		IleRS
		CysRS
		ArgRS
	Ib	GluRS
		GlnRS
		LysRS-I
	Ic	TyrRS
		TrpRS

II	IIa	SerRS
		ThrRS
		AlaRS
		GlyRS
		ProRS
		HisRS
	IIb	AspRS
		AsnRS
		LysRS-II
	IIc	PheRS

**Table 2.** Micromolar minimal inhibitory concentrations (MICs) of compounds **6**, **7** and **8**.

ID	MIC (μM)	

	*S. epidermidis* ATCC 12228	*S. epidermidis* ATCC 35984
**6**	6.25	6.25
**7**	25	25
**8**	100	100

**Table 3.** Inhibitors of threonyl-tRNA synthetase.

ID	Structure	ThrRS *K*_i_ (nM)		Selectivity ratio

		*E. coli*	Human	Human/*E.coli*
**27**	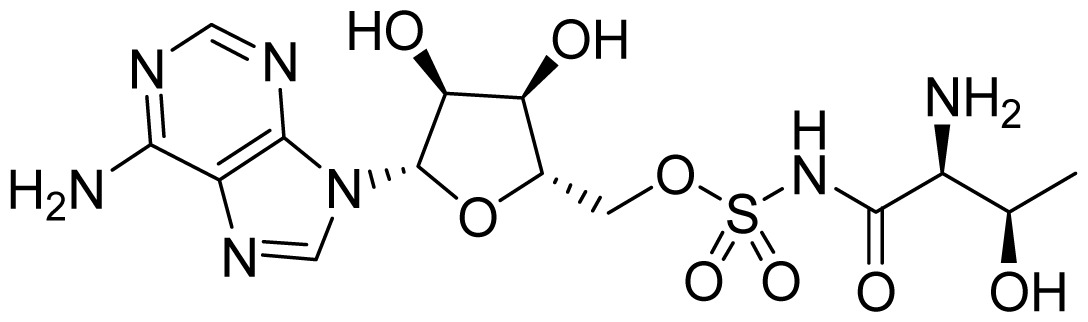	13.1	13.4	1
**28**	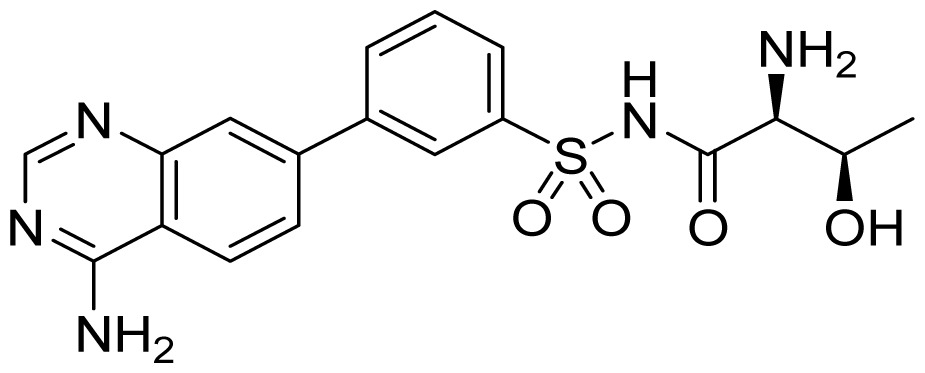	2.9	3.3	1.1
**29**	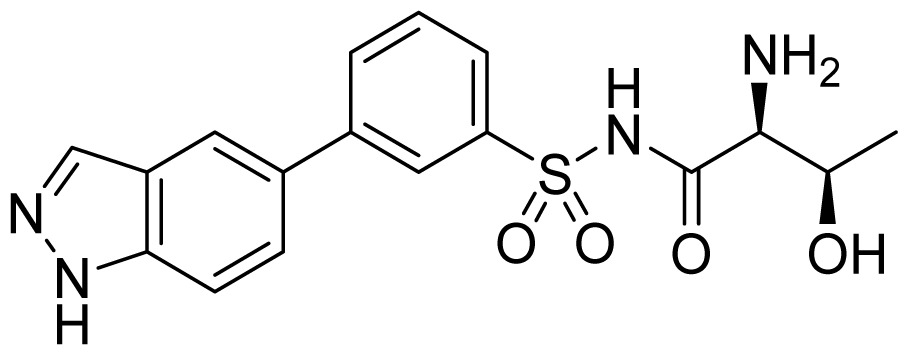	182	>50,000	>270
